# Data-derived Identity Verification as a principle for the dissemination, harmonization, and artificial intelligence reuse of sensitive biomedical data

**DOI:** 10.1093/jamiaopen/ooag087

**Published:** 2026-06-19

**Authors:** Benjamin A Cordier, Erik S Benton, Eamon J Dysinger, Shannon K McWeeney, the AI-READI Consortium, Benjamin A Cordier, Erik S Benton, Eamon J Dysinger, Shannon K McWeeney, Arash Alavi, Adrienne Baer, Amir Bahmani, Monique Bangudi, Sally L Baxter, Marian S Blazes, Steven R Chamberlin, Christopher G Chute, Jorge L Contreras, Virginia R de Sa, Nicholas Evans, Kadija Ferryman, Aydan Gasimova, Nayoon Gim, Shahin Hallaj, Anna Heinke, Stephanie Hong, Michelle R Hribar, Samm Hurst, Fritz Gerald P Kalaw, Cecilia S Lee, Aaron Y Lee, Jennifer Li- Pook-Than, Abigail Lucero, Dawn S Matthies, Gerald McGwin, Jessica Mitchell, Julie Mitchell, Liz Moyer, Camille Nebeker, Taiki Nishihara, Julia P Owen, Cynthia Owsley, Bhavesh Patel, Victoria Patronilo, Hanna Pittock, Jamie L Shaffer, Sara J Singer, Michael Snyder, Sanjay Soundarajan, Grace Walker, Brittany York, Joseph M Yracheta, Qilu Yu, Linda M Zangwill, Kevin Zhao

**Affiliations:** Knight Cancer Institute, Oregon Health & Science University, Portland, OR 97239, United States; Oregon Clinical and Translational Research Institute, Oregon Health & Science University, Portland, OR 97239, United States; Information Technology Group, Oregon Health & Science University, Portland, OR 97239, United States; Knight Cancer Institute, Oregon Health & Science University, Portland, OR 97239, United States

**Keywords:** artificial intelligence, ethical, legal, and social implications, information dissemination, data protection, Cloud Computing

## Abstract

**Objectives:**

To facilitate responsible biomedical data sharing, large-scale data harmonization, and cutting-edge artificial intelligence (AI) applications by augmenting controlled-access data dissemination models (DDMs) with Data-derived Identity Verification (DIVe).

**Materials and Methods:**

We developed a watermarking-based DIVe system that embeds a cryptographically verifiable and traceable identity within each data file. This system was deployed for the Artificial Intelligence Ready and Exploratory Atlas for Diabetes Insights Consortium and currently watermarks a 3.8TB multimodal dataset for each approved data request.

**Results:**

Over 1.8 petabytes, spanning over 900 data copies and 160 million files, have been uniquely watermarked and disseminated. Private provenance tracing, a novel data sharing license, and educational materials extend trust and accountability beyond controlled environments.

**Discussion:**

Typical controlled-access DDMs assume data remain at rest. Data-derived Identity Verification provides an organizing principle for augmenting DDMs such that data can move while maintaining protection from data leaks.

**Conclusion:**

Watermarking-based DIVe supports secure, traceable dissemination of sensitive data, supporting the development of AI technologies in biomedicine.

## The shifting biomedical data landscape

We are in a new paradigm. The rapid expansion of artificial intelligence (AI) technologies in clinical and biomedical research presents a generational opportunity to reconceive how diagnoses are made, therapies are put into practice, and health systems can efficiently deliver care of increasing quality to all.

To seize upon this opportunity and unlock the hidden value within existing biomedical data repositories, cross-study and cross-repository harmonization will often be required. For use cases requiring such harmonization, the data frequently cannot remain at rest. Yet, this fact is directly challenged by the hidden risks inherent to biomedical data; it is often sensitive, vulnerable to poorly characterized misuses (many unknowable a priori), and may be subject to export restrictions, especially to countries of concern.^1^^,^^2^ If we are to realize the potential of the opportunity before us, this tension—between the hidden risks of enabling the movement of sensitive data and the hidden value that AI technologies built upon its harmonization may unlock—must be managed. How can we protect the massive repositories of sensitive biomedical data from misuse, while also fostering a data ecosystem that enables the ethical, legal and social implications-aligned (ELSI-aligned) development of a diversity of biomedical AI and data-driven technologies?

In this work, we offer 3 contributions toward resolving this question. First, we introduce Data-derived Identity Verification (DIVe)—a generalizable, organizing principle for data dissemination models (DDMs); second, we describe its recent large-scale watermarking-based implementation for the Artificial Intelligence Ready and Exploratory Atlas for Diabetes Insights (AI-READI) Consortium; third, we discuss its role in supporting contemporary use cases, such as AI model development and equipping existing DDMs with data leak tracing capabilities. While this perspective focuses on scenarios involving the movement of biomedical data, other frameworks exist. One example is federated learning, which enables the collaborative development of foundation models by coordinating harmonization at the model layer. While federated learning is already being used in biomedical contexts and can enable an important privacy-preserving alternative for multisite AI development, it does not obviate the need for data dissemination models that support the movement and harmonization of sensitive data in settings where federated coordination is impractical or insufficient, accountability for misuse is critical, or the exploration of diverse AI model development approaches is valuable.

## Data-derived Identity Verification

The key to protecting sensitive data while also enabling its movement lies in being able to establish the provenance of any data copy by embedding within it a unique, cryptographically verifiable, and traceable identity. We term this concept Data-derived Identity Verification (DIVe). Given this capability, DDMs that enable both the harmonization necessary for AI applications and the tracing of data leaks to their source could be readily implemented.^1^^,^^3^

Conceptually, DIVe builds on forensic watermarking,^4–7^ data fingerprinting,^8–10^ and related hidden signal injection techniques. In this respect, it is an organizing principle rather than a specific technology. Examples of technologies that can implement DIVe include digital fingerprinting and client-side fully homomorphic encryption (FHE). What distinguishes DIVe from other data security measures is that the identity travels with the data regardless of how it is implemented. In practice, this means cryptographically embedding the identity of a data consumer at the data layer (ie, within the data itself). This contrasts with typical data security mechanisms for DDMs, which secure data access through identity-linked authentication at the application or operating system layer. By coupling the identity to the data, DIVe offers a complementary approach to data security for existing open- and controlled-access DDMs.^11^

To our knowledge, common controlled-access DDMs (eg, data enclaves and differential privacy) and their contemporary implementations in biomedicine currently lack a DIVe capability. This is largely due to the central assumption that the data they protect will remain at rest, with access mediated by an authentication system wrapping around the data. Historically, this assumption has comported well with the cornerstone use cases of biomedical DDMs—facilitating reproducibility research and data reuse. However, the decoupling of the data consumer’s identity from the data in these systems presents a fundamental challenge to the maintenance of ELSI-alignment (eg, through licensing) when performing cross-study and cross-repository data harmonization for AI model development. It is for this principal reason that the deployment of DIVe systems represents a key near-term goal and substantial opportunity for innovation to meet the contemporary AI paradigm.

In principle, the ideal technology to establish DIVe and enable robust data security is public-key FHE.^12^ Using a client-oriented implementation, data consumers could download, harmonize, and train AI models with an encrypted data copy in their local environment and data disseminators could maintain both traceability and a cryptographic guarantee that no sensitive or protected information would be revealed. Unfortunately, the computational overhead of existing FHE methods makes them infeasible for the large data volumes common to the biomedical domain. What is needed is a stepping stone; an approach that is sufficient, technologically feasible, and can be rolled out at pace with the rapidly evolving AI paradigm.

## History of watermarking

Digital watermarking is a data‑hiding technique in which auxiliary information, called a watermark, is imperceptibly embedded into host data such that it can later be detected or extracted, typically for purposes such as copyright protection, integrity verification, or user identification.^4–6^ Early work in multimedia watermarking established fundamental requirements—robustness to common processing, imperceptibility to human observers, and security against intentional attacks—while differentiating between robust watermarking for copyright and fragile or semifragile watermarking for authentication.^4^^,^^5^^,^^7^ Digital fingerprinting extends these ideas to code constructions that are collusion‑secure, enabling watermark traceability even when multiple watermarked data copies are combined in a collusion attack.^8–10^

In the biomedical domain, watermarking has become a key tool for securing medical images and related data in telemedicine and e‑health infrastructures, where it supports authentication, tamper detection and localization, reversible embedding in regions of nondiagnostic interest, and robust copyright or linkage of images to electronic health records while preserving diagnostic quality in the region of interest.^7^^,^^13–21^ Beyond images, recent surveys emphasize that both watermarking and fingerprinting are emerging as important mechanisms for protecting the intellectual property of AI models and building trustworthy AI‑enabled health-care systems, aligning technical protection of biomedical data and algorithms with broader governance, privacy, and accountability requirements in clinical AI deployment.^22–24^ Recent work in digital biomarkers further underscores this potential, making the case for watermarking as a means to strengthen authenticity and provenance in sensitive biomedical data streams.^25^

## DIVe into AI-READI

A central mission of the Artificial Intelligence Ready and Exploratory Atlas for Diabetes Insights (AI-READI),^26^ a data generation project of the NIH Common Fund Bridge2AI Program (B2AI), is to develop a large (estimated over 6.8TB upon completion) multimodal dataset for AI technology development. Given the sensitivity of these biomedical data, the expected harmonization work by data consumers, and established ethical mandates around data sharing and reuse, it quickly became clear that a DIVe system would be required. To meet this challenge, we developed a watermarking framework at OHSU that implements a robust form of DIVe for the AI-READI DDM with the following core capabilities:

1. The provisioning of a secure, unique, and cryptographically verifiable identity encoded as a nonintrusive, imperceptible watermark within each file of each data copy disseminated to each data consumer.
2. The private tracing of any watermark within the data copy, such that the unique identity of the data consumer can be revealed by the data disseminator.

Building the DIVe-enabled DDM for AI-READI involved the development and deployment of a Cloud-based data pipeline,^27^ which currently automates the watermarking of over 356 000 multimodal (tabular, waveform, and imaging) data files (3.8TB) for each approved data request.

Separately, we have developed a watermarking library that includes extensions of established digital fingerprinting codes^10^^,^^11^ and a built-in validation suite that uses simulation-based robustness testing, including collusion, boundary, and noise injection attacks,^8^^,^^10^^,^^28^ to verify that the system configurations maintain their intended cryptographic and traceability guarantees. Multiple watermarking algorithms and parameterizations are implemented as part of the AI-READI DDM, each targeted and validated against the specific data modality. These watermarks exist at both global and local levels, enabling the tracing of individual files and, in many cases, file fragments or subsets (eg, rows, columns, image patches). As a consequence, subsets of a watermarked file may remain traceable provided sufficient signal is retained. In principle, below a threshold (varying by modality, algorithm, and parameterization), watermark traceability may degrade. This is a known limitation of the system and inherent to the nature of watermarking generally and digital fingerprinting techniques in particular. Full details of the algorithms, parameterizations, and cryptographic verification, including their validation on the AI-READI’s dataset, will be provided in a forthcoming paper.

Prior to the download of protected data on Fairhub.io, data consumers are educated on the required data stewardship, consent to the use of the DIVe system to trace any unsanctioned release of their data copy, and legally attest that they will not violate the terms of the AI-READI license,^29^ a novel license developed by the AI-READI DGP in collaboration with legal scholars. Once their request is approved, the watermarking procedure is initiated and the downloader is notified when their uniquely watermarked data copy is ready for download. Together, this combination of education, consent, and legal attestation is critical to supporting the ELSI-aligned data analysis, reproducibility, and AI model development by consumers of the AI-READI dataset.^30^

While the DIVe-enabled AI-READI DDM does not prevent the unsanctioned release of data that is human-readable, it does provide a cryptographically secured method for tracing the origin of any unsanctioned release of a data copy, which provides a measure of protection against misuse and accountability for breaches of licensing. It is for this key reason that DIVe is well suited to DDMs disseminating sensitive data without PHI—it enables reactive accountability via the tracing of leaks, rather than proactive prevention, and is insufficient on its own to meet the legal and ethical requirements for PHI data stewardship. Nonetheless, for DDMs that do provide access to PHI, DIVe may offer a complement to existing preventive controls (eg, encryption, deidentification, and highly secured, HIPAA-compliant environments), contributing to a “defense-in-depth” cybersecurity model.

Security tools that complement existing PHI protection systems are much needed. Within health care, there exists an ongoing deluge of data breaches affecting health-care organizations. At the time of writing, the Department of Health & Human Services is investigating PHI breaches affecting 909 organizations and an estimated 170 million individual records from the past 24 months.^3^ While “Hacking/IT Incident” is the most common class of data breach (760 incidents), “Unauthorized Access/Disclosure” is the second-most common classification (120 incidents). This comports with other studies, for example,^1^ which indicate that breaches can often be sourced to human error. In the context of data breaches, it is those that can be attributed to human factors for which DIVe—whether by watermarking, homomorphic encryption, or some other technology—may prove most valuable.

Finally, while motivated by AI-READI, the watermarking library has been built as a modular, and context-agnostic software library that can operate as a composable add-on to existing DDMs, such as data enclaves. Crucially, it includes a built-in simulation suite for creating, attacking, and tracing watermarks to ensure implementations maintain cryptographic guarantees. In this respect, we hope that our forthcoming watermarking library will offer the opportunity for data disseminators to augment existing DDM implementations with the fine-grained, user-level, DIVe required to enable the tracing of any data breach to its source and—for data licensed specifically for AI reuse—enable ELSI-aligned data dissemination.

## Looking to the future

As we progress further into the novel AI paradigm, it is our hope that DIVe can serve as an organizing principle for sensitive biomedical data dissemination and that DIVe implementations, such as AI-READI’s, will enable the traceable movement of sensitive biomedical data, thus facilitating the harmonization work of data consumers critical to the development of frontier AI technologies in biomedicine.

## Supplementary Material

ooag087_Supplementary_Data

## Data Availability

Data used for this work are available from https://fairhub.io, a platform developed for the dissemination of findable, accessible, interoperable, and reproducible data.
